# Maternal health care professionals’ experiences and views on the use of obstetric ultrasound in Rwanda: A cross-sectional study

**DOI:** 10.1186/s12913-021-06758-w

**Published:** 2021-08-10

**Authors:** Ingrid Mogren, Joseph Ntaganira, Jean Paul Semasaka Sengoma, Sophia Holmlund, Rhonda Small, Lan Pham Thi, Hussein Lesio Kidanto, Matilda Ngarina, Cecilia Bergström, Kristina Edvardsson

**Affiliations:** 1 Department of Clinical Sciences, Obstetrics and Gynecology, Umeå University, S-90187 Umeå, Sweden; 2 Judith Lumley Centre, School of Nursing and Midwifery, La Trobe University, Melbourne, Australia; 3 School of Public Health, College of Medicine and Health Sciences, University of Rwanda, Kigali, Rwanda; 4 Department of Nursing, Umeå University, Umeå, Sweden; 5 Department of Women’s and Children’s and Reproductive Health, Karolinska Institutet, Stockholm, Sweden; 6 Department of Dermatology and Venereology, Hanoi Medical University, Hanoi, Vietnam; 7 Medical College, East Africa Aga Khan University, Dar es Salaam, Tanzania; 8 Department of Obstetrics and Gynaecology, Muhimbili University of Health and Allied Sciences, Dar es Salaam, Tanzania

**Keywords:** Rwanda, Ultrasonography, Obstetrics, Pregnancy, Health professionals, Obstetricians, Gynecologists, Midwives, Nurses, Questionnaire, Epidemiology, Commercialisation, Clinical guidelines, Medicalisation

## Abstract

**Background:**

This study, undertaken in Rwanda, aimed to investigate health professionals’ experiences and views on the following topics: current clinical guidelines for ultrasound from second trimester at the clinic, regional and national levels, and adherence to clinical guidelines; medically indicated ultrasound examinations; non-medical use of ultrasound including ultrasounds on maternal request; commercialisation of ultrasound; the value of ultrasound in relation to other clinical examinations in pregnancy; and ultrasound and medicalisation of pregnancy.

**Methods:**

A cross-sectional design was adopted. Health professionals providing antenatal care and delivery services to pregnant women in 108 health facilities were invited to complete a survey, which was developed based on the results of earlier qualitative studies undertaken as part of the CROss Country Ultrasound Study (CROCUS).

**Results:**

Nine hundred and seven health professionals participated: obstetricians/gynecologists (3.2%,) other physicians (24.5%), midwives (29.7%) and nurses (42.7%). Few physicians reported the existence of clinical guidelines at clinic, regional or national levels in Rwanda, and guidelines were moderately adhered to. Three obstetric ultrasound examinations were considered medically indicated in an uncomplicated pregnancy. Most participants (73.0%) were positive about obstetric ultrasound examinations on maternal request. Commercialisation was not considered a problem, and the majority (88.5%) agreed that ultrasound had contributed to medicalisation of pregnancy.

**Conclusions:**

Findings indicate that clinical guidelines for the use of obstetric ultrasound are limited in Rwanda. Non-medically indicated obstetric ultrasound was not considered a current problem at any level of the healthcare system. The positive attitude to obstetric ultrasound examinations on maternal request may contribute to further burden on a maternal health care system with limited resources. It is essential that limited obstetric ultrasound resources are allocated where they are most beneficial, and clearly stated medical indications would likely facilitate this.

## Background

Ultrasound has become an essential part of pregnancy management across the globe. The World Health Organization (WHO) recommends one ultrasound scan before 24 weeks of gestation for all pregnant women primarily to estimate gestational age, determine placental location and single or multiple pregnancy, improve detection of fetal anomaly, and to enhance the pregnancy experience [1]. In many high-income countries such as the United States, the UK and Australia [2–4], in addition to the recommended standard examination in the second trimester [2], an early scan in the first trimester is increasingly used for confirmation of viability, assessment of gestational age and identification of multiple pregnancy, often combined with screening for fetal chromosomal anomalies [5]. Diagnostic ultrasound is an important tool in the investigation of signs of complications, including concerns about fetal growth and well-being [1]. In high-risk pregnancies, Doppler ultrasound has been shown to reduce the risk of perinatal death and prevent unnecessary obstetric interventions [6].

Ultrasound has increasingly become an indispensable part of pregnancy management in many low-income settings [7], although access may vary widely between urban and rural areas [7, 8]. Common barriers to the use of ultrasound include availability of electricity, lack of technical support and repair, as well as lack of training opportunities for health professionals [7, 9, 10]. Evidence regarding the value of routine ultrasound in these settings is conflicting [11]. Some evidence suggests that introduction of routine ultrasound examinations has benefits including increased use of ANC, deliveries at health facilities and referrals for obstetric complications [12–14]. However, in a recent cluster randomised trial in five countries including Democratic Republic of Congo, Guatemala, Kenya, Pakistan, and Zambia, introduction of two routine ultrasound scans at 16–22 weeks and 32–36 weeks, did not increase ANC attendance or hospital delivery for complicated pregnancies, and did not improve maternal, fetal and neonatal mortality, or near-miss maternal mortality. The authors concluded that introduction of routine ultrasound without an overall improvement in the quality of care has limited effect in low- and middle-income countries [15]. Nevertheless, a number of studies have indicated important benefits of selective obstetric ultrasound in these settings, including confirmation of clinically suspected obstetric complications, improved patient management and quality of care [11].

Rwanda is a landlocked country in East-Central Africa, with a population of 12.6 million. Since the 1994 genocide against the Tutsi, the country has seen substantial progress in population health, and between 1990 and 2015 maternal mortality decreased by 78% [16]. This improvement has been attributed to the government’s commitment to improve reproductive, maternal, newborn and child health, and the introduction of a community-based health insurance scheme which has ensured access to maternal and child health services for vulnerable populations [16]. Today, the majority (93%) of live births are delivered in health facilities (the majority in local health centres) and 94% are assisted by skilled health providers, mainly nurses [17].

The Rwandan Ministry of Health (MOH) recommends at least four antenatal care visits during pregnancy. The latest national Demographic and Health Survey from 2019/2020 reports that 99% of women attend at least one visit, while 47.2% of women attend the recommended four visits, [17]. There is a shortage of physicians in Rwanda, although women who live in urban areas, who have higher levels of education and are financially better off, are more likely to consult a physician during pregnancy [17]. Many women (59%) experience serious barriers to accessing health care, most commonly financial barriers and distance to health facilities, particularly for women in rural areas [18].

Ultrasound examinations are not yet a routine part of antenatal care in Rwanda, however we have reported from a previous qualitative study that health care providers in Rwanda place a high value on ultrasound in pregnancy management, and also that they experience increasing demand for ultrasound from pregnant women [9]. Access is still limited at lower levels of care, particularly in health care centres where the majority of women give birth, with very limited access in rural areas. There are indications of biased allocation of resources between socioeconomic groups [8, 9] since private clinics may offer more generous access, also on maternal request [9]. The majority of health professionals caring for pregnant women in Rwanda consider ultrasound as vital in pregnancy management and are supportive of routine use [8]. At the same time, professionals have expressed concerns that women may perceive ultrasound as superior to other fundamental clinical examinations in pregnancy [9].

With increasing access and availability of ultrasound in a low-income setting like Rwanda, it seems important to explore the role of ultrasound in clinical management of pregnancy from health professionals’ perspectives, in order to safeguard appropriate use and continuous improvement in health care for pregnant women.

The overall aim of this study was to investigate health professionals’ experiences and views on different aspects of obstetric ultrasound in Rwanda. The topics of interest were *1) Current clinical guidelines for ultrasound from the second trimester at the clinic, regional and national levels, and adherence to clinical guidelines; 2) Views on medically indicated ultrasound examinations; 3) Non-medical use of ultrasound (*i.e. *obstetric ultrasound examinations during pregnancy without medical indication) including ultrasounds on maternal request; 4) Commercialisation of ultrasound; 5) The value of ultrasound in relation to other clinical examinations in pregnancy; and 6) Views on use of ultrasound and medicalisation of pregnancy.*

## Methods

### Study setting

The study setting of Rwanda has been extensively described in a previous publication [8]. Briefly, the Rwandan population is estimated at approximately 12.6 million, the health system structure is pyramidal with health posts and health centres at the bottom and referral hospitals at the top, and the number of births annually are around 310,000 [8]. The study adopted a cross-sectional study design using questionnaires with obstetricians/ gynecologists/physicians and midwives/nurses providing antenatal care and delivery services to pregnant women in Rwanda.

### Selection of health facilities

The selection of health facilities has been described in detail in a previous publication [8]. In summary, the study included health facilities from all four provinces of Rwanda (North, East, South, and West) and the area of Kigali city. All provincial hospitals (*n* = 4) and referral hospitals (*n* = 7), the largest private hospitals (*n* = 12), 20 district hospitals, and 65 health centres were included in the study, to ensure inclusion of health facilities at all levels in Rwanda [8].

### Sample size and study participants

The sample size was based on estimations of the prevalence of background and outcome variables [8]. Health professionals with different experiences of obstetric ultrasound, either working with ultrasound examinations as a major part of their duties, *or* performing ultrasound examinations as part of their general obstetric care, *or* using the results of ultrasound in clinical management of pregnant women, were eligible participants for the study (obstetricians/gynecologists, other physicians, midwives and nurses). Health professionals at health centres were also included in the study, to obtain additional experiences, although they rarely accessed or performed obstetric ultrasound themselves. Radiology staff were excluded since they are not primary care providers for pregnant women. The final sample consisted of 907 participants [8]. Contact with the study sites was initiated by authors JN and JPS, and data collection was undertaken by trained data collectors (three nurses and one clinical officer) between November 2016 and March 2017. The data collectors visited all study sites and invited all obstetricians/physicians and midwives/nurses working on the day of the data collection to take part in the study.

### The data collection tool – a multifaceted questionnaire

The research team developed a questionnaire based on the results of the earlier qualitative studies performed in the CROss Country Ultrasound Study (CROCUS) [9, 10, 19–24]. The questionnaire included 105 different items. Examples of items analysed in this article are socio-demographic characteristics, guidelines for ultrasound use including clinical guidelines, statements on ultrasound resources, and technical developments in maternity care. Most questions, statements and their response options are presented in Table 1 below. Participants were not asked about whether they had received any formal training in obstetric ultrasound. The development of the questionnaire has been reported in detail elsewhere [8]. In summary, it was initially developed in English and thereafter translated to French, because medical terms used in Rwandan hospitals are commonly in French. The questionnaire was pilot-tested at two different hospitals in Rwanda by 20 health professionals. As a result of the pilot, the questionnaire was also translated into Kinyarwanda. This language-version was also pilot-tested resulting in only minor changes. Since Rwanda is a multilingual country, a decision was taken to provide the participants with the opportunity of responding to the questionnaire in either Kinyarwanda, French or English. Most participants chose to answer the questionnaire in Kinyarwanda, followed by French and English.

**Table 1:** Questions and statements and their response options in the questionnaire

*Are there any guidelines at your clinic/work place for use of ultrasound in pregnancy from the second trimester?*	
• Clinic guidelines^a^	
• Regional guidelines^a^	
• National guidelines^a^	
*From your own experience, to what extent are these guidelines followed?*	
• Clinic guidelines^b^	
• Regional guidelines^b^	
• National guidelines^b^	
*In your view, how many ultrasound examinations are medically indicated in an uncomplicated pregnancy?*	
• Number of ultrasounds	
*Statements about the use of ultrasound*	
• Obstetric ultrasound examinations are often performed for non-medical purposes in my country^c^	
• Pregnant women should be able to have non-medical ultrasounds on their own request^c^	
• Commercialisation of ultrasound is a problem in my country^c^	
• Commercialisation of ultrasound is a problem in my hospital/clinic^c^	
• Do you feel that pregnant women expect to have an ultrasound during consultations, even when there is no medical indication for ultrasound?^c^	
*Statements on technical developments in maternity care*	
• Maternity care providers may trust ultrasound above clinical examinations in pregnancy^c^	
• Increasing use of obstetric ultrasound may result in less focus on clinical skills^c^	
• The use of ultrasound has contributed to medicalisation of pregnancy^c^	

^a^Response options: Yes, No, Don’t know

^c^Response options: Strongly agree, Agree, Neutral, Disagree, Strongly disagree

^b^Response options: Don't know, Not at all, To a small extent, To a moderate extent, To a great extent

### Statistics

The data were analysed with descriptive statistics. Differences in mean values were assessed using the Student’s t-test, and Pearson’s Chi-Square test was used for categorical differences, with a *p*-value of 0.05 for statistical significance. Venn diagrams have also been used to illustrate similarities and differences in agreement/disagreement with selected statements. Univariable and multivariable logistic regression analyses were undertaken for selected exposures and outcomes, calculating odds ratios (OR) and their 95% confidence intervals (CI) for associations. When performing univariable and multivariable logistic regression analysis for different statements originally including five response options, the response option “neutral” was always excluded from the analysis. Pearson’s correlation coefficient (r^2^) was calculated when applicable. SPSS version 27 was used for all analyses.

## Results

In total, 907 health professionals participated in the study including the following health professional categories: obstetricians/gynecologists (OG) 3.2% (*n* = 29), other physicians (OP) 24.5% (*n* = 222), midwives 29.7% (*n* = 269) and nurses 42.7% (*n* = 387). Table 2 presents the background characteristics for the sample. Categorizing the health professionals in relation to health profession and workplace showed that 27.7% (*n* = 251) of participants were physicians working in hospitals (P-H); 36.7% (*n* = 333) of participants were midwives/nurses working in hospitals (MN-H; the majority were midwives); and 35.6% (*n* = 323) were nurses/midwives working in health centres (NM-HC; the majority were nurses) (Table 2).

**Table 2:** Background characteristics of the study sample (*N* = 907)

Variable^a^	All health professionals	Physicians in hospitals (P-H)	Midwives/nurses in hospitals (MN-H)	Nurses/midwives in health centres (NM-HC)
	N = 907 (100%)	n = 251 (27.7%)	n = 333 (36.7%)	n = 323 (35.6%)
	**n (%)**	**n (%)**	**n (%)**	**n (%)**
Gender	*907 (100)*	*251 (100)*	*333 (100)*	*323 (100)*
Male	358 (39.5)	189 (75.3)	59 (17.7)	110 (34.1)
Female	549 (60.5)	62 (24.7)	274 (82.3)	213 (65.9)
Age, years	*904 (99.7)*	*248 (98.8)*	*333 (100)*	*323 (100)*
Mean; SD^b^	35.0; 7.8	33.7; 9.1	34.6; 7.1	36.5; 8.1
Min-Max	21–68	22–68	22–60	21–68
Marital status	*905 (99.8)*	*251 (100)*	*332 (99.7)*	*322 (99.7)*
Married	619 (68.2)	123 (49.0)	257 (77.4)	239 (74.2)
Cohabiting	10 (1.1)	–	1 (0.3)	9 (2.8)
Separated/Divorced	4 (0.4)	–	3 (0.9)	1 (0.3)
Widowed	19 (2.1)	3 (1.2)	6 (1.8)	10 (3.1)
Not married/Single	253 (28.0)	125 (49.8)	65 (19.6)	63 (19.6)
Having children	*894 (98.6)*	*248 (98.8)*	*328 (98.5)*	*318 (98.5)*
Yes	615 (68.8)	112 (45.2)	251 (76.5)	252 (79.2)
No	279 (31.2)	136 (54.8)	77 (23.5)	66 (20.8)
Years in profession	*907 (100)*	*251 (100)*	*333 (100)*	*323 (100)*
Mean; SD^b^	6.3; 6.2	4.7; 6.1	5.1; 4.1	8.8; 7.2
Min-max	0–44	0–39	0–27	0–44
Years in health care	*905 (99.8)*	*250 (99.6)*	*333 (100)*	*322 (99.7)*
Mean; SD^b^	8.9; 7.3	6.1; 6.8	9.2; 6.6	10.7; 7.7
Min-max	0–44	0–39	0–39	0–44
Public/private health care	*904 (99.7)*	*250 (99.6)*	*331 (99.4)*	*323 (100)*
Public	702 (77.7)	199 (79.6)	245 (74.0)	258 (79.9)
Private	71 (7.9)	23 (9.2)	44 (13.3)	4 (1.2)
Both public and private	131 (14.5)	28 (11.2)	42 (12.7)	61 (18.9)
Area of health facility	*907 (100)*	*251 (100)*	*333 (100)*	*323 (100)*
Kigali^c^	283 (31.2)	80 (31.9)	133 (39.9)	70 (21.7)
Other areas^d^	624 (68.8)	171 (68.1)	200 (60.1)	253 (78.3)
Provision of maternity services^e^				
Antenatal care	647 (71.3)	194 (77.3)	177 (53.2)	276 (85.4)
Intrapartum care	775 (85.4)	227 (90.4)	275 (82.6)	273 (84.5)
Postpartum care	722 (79.6)	217 (86.5)	254 (76.3)	251 (77.7)
Do not currently provide maternity care	70 (7.7)	22 (8.8)	26 (7.8)	22 (6.8)
Performing ultrasound^f^	*906 (99.9)*	*250 (99.6)*	*333 (100)*	*323 (100)*
Yes	293 (32.3)	239 (95.6)	54 (16.2)	0
No	613 (67.7)	11 (4.4)	279 (83.8)	323 (100)

^a^The denominator in all calculations is the total number included in each category of health professional

^b^SD = Standard Deviation

^c^All levels of health facilities in the area around Kigali (n = 29)

^d^All levels of health facilities in the area outside Kigali (*n* = 79)

^e^Item in questionnaire: “Which of the following maternity services do you provide? (Please tick all that apply)”

^f^Performing ultrasound examinations

### Clinical guidelines

Participating hospital physicians and midwives/nurses reported the existence of clinical guidelines on use of ultrasound from the second trimester at clinic, regional and national levels in Rwanda in the following proportions, 11.0% (*n* = 49), 7.2% (*n* = 18) and 25.5% (*n* = 64), respectively. Non-existence of clinical guidelines for these three health care levels was reported by participating physicians as 41% (*n* = 103), 50% (*n* = 125) and 52.6% (*n* = 132), respectively. Forty-five percent (113/251) of physicians responded to the question whether clinical guidelines were followed at their workplace. Of these, 40.7% responded (46/113) “don’t know”, and 15 % “not at all” or “to a small extent”. A proportion of 44.2% replied that guidelines were followed “to a moderate extent” or “to a great extent”.

### Number of ultrasound examinations medically indicated in an uncomplicated pregnancy

The reported mean number of medically indicated ultrasound examinations during an uncomplicated pregnancy was 3.2 across all health professionals (*n* = 898; range 0–10; SD 1.25). The mean number of medically indicated ultrasound examinations as reported by P-H, MN-H and NM-HC was 3.7, 3.3, and 2.8, respectively. Comparing different health professional categories, there were statistically significant differences in mean numbers (3.7 vs. 3.3; t-test *p* < 0.001; 3.7 vs. 2.8; t-test p < 0.001). Participants performing ultrasound examinations reported more on average as medically indicated, compared with those not themselves performing ultrasounds (3.6 vs 3.0; p < 0.001). There were weak negative correlations between the mean number of indicated ultrasounds and participant age (r^2^ = − 0.083; *p* = 0.013), and years in health care (r^2^ = − 0.068; *p* = 0.043). Mean numbers for medically indicated ultrasound during an uncomplicated pregnancy were similar between health professionals working solely in public health care (3.2), in private health care (3.3), or in both public and private health care (3.1).

Responses to the pre-specified statements shown in Table 1 are presented below and in Table 3. In the text following, the term “agreed” combines response options “agree” and “strongly agree”. The term “disagreed” is used in the same way.

**Table 3:** Health professionals’ responses^a^ to statements about ultrasound by background characteristics and other selected variables

**Background variables**	*Obstetric ultrasound examinations are often performed for non-medical purposes in my country*			*Pregnant women should be able to have non-medical ultrasound on their own request*		
	Agree or strongly agree	Disagree or strongly disagree	*p*-value^b^	Agree or strongly agree	Disagree or strongly disagree	*p*-value^b^
	**n (%)**	**n (%)**		**n (%)**	**n (%)**	
**Health professional/workplace (*n*)**	***(901)***			***(903)***		
Physicians in hospitals	26 (10.4)	166 (66.4)	***0.001***	165 (65.7)	43 (17.1)	***0.049***
Midwives/nurses in hospitals	46 (13.9)	245 (73.8)		240 (72.7)	61 (18.5)	
Nurses/midwives in health centres	44 (13.8)	256 (80.3)		254 (78.9)	57 (17.7)	
**Age (*n*)**	***(898)***			***(900)***		
≤ 35 years	64 (12.3)	386 (73.9)	*0.727*	377 (71.8)	92 (17.5)	*0.313*
> 35 years	51 (13.6)	279 (74.2)		281 (74.9)	68 (18.1)	
**Public/Private health care**^c^**(*n*)**	***(898)***			***(901)***		
Public	85 (12.2)	514 (73.7)	*0.386*	513 (73.2)	118 (16.8)	***0.036***
Private	10 (14.3)	49 (70.0)		49 (69.0)	16 (22.5)	
Both public and private	20 (15.3)	103 (78.6)		95 (73.6)	27 (20.9)	
**Public/Private health care**^d^**(*n*)**	***(898)***			***(901)***		
Public	85 (12.2)	514 (73.7)	*0.322*	513 (73.2)	118 (16.8)	***0.003***
Private but also public	30 (14.9)	152 (75.6)		144 (72.0)	43 (21.5)	
**Performing ultrasound**^e^**(*n*)**	***(900)***			***(902)***		
Yes	29 (9.9)	199 (68.2)	***0.001***	196 (67.1)	51 (17.5)	***0.001***
No	87 (14.3)	467 (76.8)		462 (75.7)	110 (18.0)	
**Indicated ultrasound exam. in uncomplicated pregnancy**^f^	***(893)***			***(895)***		
≤ 3	85 (13.8)	463 (74.9)	*0.051*	458 (74.1)	109 (17.6)	*0.390*
> 3	30 (10.9)	199 (72.4)		198 (71.5)	50 (18.1)	
**“Ultrasound is safe to use”**^g^**(*n*)**	***(788)***			***(791)***		
Agree or strongly agree	88 (12.5)	526 (74.6)	*0.906*	525 (74.0)	120 (16.9)	*0.108*
Disagree or strongly disagree	10 (12.0)	62 (84.7)		52 (63.4)	23 (28.0)	
**“Medicalisation of pregnancy**^h^**(*n*)**	***(810)***					
Agree or strongly agree	101 (13.8)	544 (74.5)	*0.161*	548 (74.9)	130 (17.8)	*0.118*
Disagree or strongly disagree	6 (7.5)	60 (75.0)		53 (66.3)	17 (21.3)	
	*Commercialisation of ultrasound is a problem in my country*			*Commercialisation is a problem in my hospital/clinic*		
	Agree or strongly agree	Disagree or strongly disagree	*p*-value^b^	Agree or strongly agree	Disagree or strongly disagree	*p*-value^b^
	**n (%)**	**n (%)**		**n (%)**	**n (%)**	
**Health professional/workplace (*n*)**	***(900)***			***(896)***		
Physicians in hospitals	40 (16.1)	116 (46.4)	***0.001***	24 (9.7)	145 (58.5)	***0.001***
Midwives/nurses in hospitals	47 (14.2)	240 (72.7)		29 (8.8)	267 (81.2)	
Nurses/midwives in health centres	51 (15.9)	240 (74.8)		28 (8.8)	261 (81.8)	
**Age (*n*)**	***(897)***			***(893)***		
≤ 35 years	87 (16.6)	323 (61.5)	***0.003***	48 (9.2)	380 (73.1)	*0.321*
> 35 years	51 (13.7)	271 (72.8)		33 (8.8)	291 (78.0)	
**Public/Private health care**^c^**(*n*)**	***(897)***			***(893)***		
Public	111 (15.9)	447 (64.2)	*0.401*	65 (11.4)	504 (88.6)	*0.110*
Private	8 (11.4)	51 (72.9)		5 (8.1)	57 (91.9)	
Both public and private	19 (14.5)	96 (73.3)		10 (7.6)	111 (84.7)	
**Public/Private health care**^d^**(*n*)**	***(897)***			***(893)***		
Public	111 (15.9)	447 (64.2)	*0.133*	65 (9.4)	504 (72.9)	***0.018***
Private but also public	27 (13.4)	147 (73.1)		15 (7.4)	168 (83.2)	
**Performing ultrasound**^e^**(*n*)**	***(899)***			***(895)***		
Yes	49 (16.8)	146 (50.2)	***0.001***	27 (9.3)	182 (62.8)	***0.001***
No	89 (14.6)	449 (73.8)		54 (8.9)	490 (81.0)	
**Indicated ultrasound exams in uncomplicated pregnancy**^f^	***(892)***			***(888)***		
≤ 3	101 (16.4)	421 (68.2)	***0.006***	56 (9.1)	475 (77.5)	***0.010***
> 3	36 (13.1)	171 (62.2)		24 (8.7)	192 (69.8)	
**“Ultrasound is safe to use”**^g^**(*n*)**	***(787)***			***(786)***		
Agree or strongly agree	112 (15.9)	456 (64.6)	*0.286*	68 (9.7)	522 (74.1)	*0.691*
Disagree or strongly disagree	12 (14.8)	61 (75.3)		6 (7.3)	66 (80.5)	
**“Medicalisation of pregnancy**^h^**(*n*)**	***(809)***			***(806)***		
Agree or strongly agree	113 (15.5)	497 (68.2)	*0.942*	67 (9.2)	557 (76.5)	*0.151*
Disagree or strongly disagree	12 (15.0)	56 (70.0)		6 (7.7)	60 (76.9)	
	*Do you feel that pregnant women expect to have an ultrasound during consultations, even when there is no medical indication for ultrasound?*					
	Agree or strongly agree	Disagree or strongly disagree				
	**n (%)**	**n (%)**				
**Health professional/workplace (*n*)**	***(906)***					
Physicians at hospitals	182 (72.5)	45 (17.9)	***0.021***			
Midwives/nurses at hospitals	202 (60.7)	89 (26.7)				
Nurses/midwives at health centres	174 (54.0)	118 (36.6)				
**Age (*n*)**	***(903)***		*0.587*			
≤ 35 years	331 (62.8)	137 (26.0)				
> 35 years	224 (59.6)	115 (30.6)				
**Public/Private health care**^c^**(*n*)**	***(903)***					
Public	443 (63.2)	179 (25.5)	***0.015***			
Private	33 (46.5)	29 (40.8)				
Both private and public	79 (60.3)	44 (33.6)				
**Public/Private health care**^d^**(*n*)**	***(903)***					
Public	443 (63.2)	179 (25.5)	***0.022***			
Private but also public	112 (55.4)	73 (36.1)				
**Performing ultrasound**^e^**(*n*)**	***(905)***					
Yes	203 (69.3)	60 (20.5)	***0.011***			
No	354 (57.8)	192 (31.4)				
**Indicated ultrasound exams in uncomplicated pregnancy**^f^	***(898)***					
≤ 3	370 (59.7)	183 (29.5)	*0.349*			
> 3	181 (65.1)	68 (24.5)				
**“Ultrasound is safe to use”**^g^**(*n*)**	***(793)***					
Agree or strongly agree	455 (64.1)	182 (25.6)	***0.002***			
Disagree or strongly disagree	39 (47.0)	38 (45.8)				
**“Medicalisation of pregnancy**^h^**(*n*)**	***(815)***					
Agree or strongly agree	467 (63.5)	198 (26.9)	*0.614*			
Disagree or strongly disagree	46 (57.5)	26 (32.5)				

^a^All five categories of response were included as separate categories in analysis: “Strongly agree”, “Agree”, “Neutral”, “Disagree”, “Strongly disagree”. Response option “neutral” not presented in the table

^b^Pearson’s Chi-Square test for comparison of difference between categories

^c^Included in analysis are only participants who reported working either in public or private health care

^d^Included in analysis are participants who reported working either in public health care solely or working in both public and private health care

^e^Performing ultrasound examinations

^f^Responses to question: “In your view, how many ultrasound examinations are medically indicated in an uncomplicated pregnancy”?

^g^Pre-specified statement: “Ultrasound is safe to use for the pregnant woman and the fetus irrespective of the number of examinations”. Nine hundred and three participants responded to this question. Results on this statement have been previously published [1]

^h^Pre-specified statement: “The use of ultrasound has contributed to medicalisation of pregnancy”. Nine hundred and three participants responded to this question. Results for this statement have been previously published [1]

### Obstetric ultrasound examinations are often performed for non-medical purposes in my country

Most (74.0%) participants disagreed with the statement, with 12.9% agreeing (Table 3). There was a significant difference between those performing ultrasound examinations and those not (*X*^*2*^; *p* = 0.001). There was also a difference of opinion by health profession/workplace (*X*^*2*^; p = 0.001), where physicians in hospitals were “neutral” (23.2%) to a greater extent than midwives/nurses in hospitals (12.3%) and nurses/midwives in health centres (6.0%). When excluding “neutral” responses in logistic regression analysis however, there was no significant difference between groups (ie. agree/strongly agree vs. disagree/strongly disagree) for either health profession/workplace or whether performing ultrasound or not.

### Pregnant women should be able to have non-medical ultrasound at their own request

A high proportion (73.0%) of all participants agreed with the statement, with 17.8% disagreeing (Table 3). Midwives and nurses in hospitals and health centres agreed to a greater extent with the statement compared with physicians in hospitals (Table 3; *X*^*2*^; *p* = 0.049). When excluding the response option “neutral” in analyses and categorizing midwives/nurses in one group and physicians as the reference group the crude odds ratio was not statistically significant (*n* = 820). Participants performing ultrasound examinations agreed to a lesser extent with the statement when compared with participants not performing ultrasound (*X*^*2*^; *p* = 0.001), however when excluding neutral responses in analysis, the odds ratio was not statistically significant (*n* = 819). The proportions of female and male physicians agreeing with the statement were 83.6% vs. 77.9% respectively, however non-significant in (*X*^*2*^; *p* = 0.357).

### Commercialisation of ultrasound is a problem in my country

A majority of all participants (66.2%) disagreed with the statement, with 15.3% agreeing. The proportions of different health professionals agreeing with the statement were similar, ranging from 14.2 to 16.1% (Table 3). When excluding the response option “neutral” in analyses and comparing physicians with midwives/nurses at hospitals, physicians disagreed to a lesser extent with the statement when compared to midwives/nurses at hospitals (crude odds ratio; COR 0.57; CI 0.35–0.91; *n* = 443). Age was associated with agreement or disagreement with the statement (*p* = 0.003; Table 3). There was a close to significant increased odds ratio of the higher age group (≥35 years) disagreeing (COR 1.43, CI 0.977–2.10; *p* = 0.065; *n* = 732) when compared to younger participants (<35 years). Whether performing ultrasound or not was associated with the statement (*X*^*2*^; *p* = 0.001). When excluding the response option “neutral”, participants not performing ultrasound were more likely to disagree with the statement (COR 1.69; CI 1.13–2.51; *n* = 733) when compared to participants performing ultrasound.

### Commercialisation of ultrasound is a problem in my hospital/clinic

Most participants disagreed (75.1%) with this statement, with a small minority (9.0%) agreeing. When excluding the response option “neutral”, none of the statistically significant associations in Table 3 remained significant.

### Pregnant women expect to have an ultrasound during consultations, even when there is no medical indication for ultrasound

More than half of all participants (61.6%) agreed with this statement, with 27.8% disagreeing. Physicians in hospitals were more likely to agree with the statement (COR 1.78, CI 1.18–2.69, *n* = 518) when compared to midwives/nurses in hospitals. Participants performing ultrasound were also more likely to agree with the statement when compared to participants not performing ultrasound (COR 1.83, CI 1.31–2.58; *n* = 809). Participants who agreed with the statement that “Ultrasound is safe to use for the pregnant woman and her fetus irrespective of the number of examinations” were also more likely to agree with the above statement (COR 2.44, CI 1.50–3.94; *n* = 714).

### Maternity care providers may trust ultrasound above clinical examinations in pregnancy

More than half (57.6%) of all participants agreed with this statement, with 32.6% disagreeing (Table 4). There was a statistically significant difference between health professional categories (*X*^*2*^*; p* = 0.014; Table 4) as well as for the two categories performing or not performing ultrasound (*X*^*2*^; *p* = 0.001; Table 4). When excluding the response option “neutral” from analysis, the significant associations became non-significant for the two background variables health profession category and whether performing ultrasound or not. Participants agreeing with the statement “*Pregnant women expect to have an ultrasound during consultations, even when there is no medical indication for ultrasound*” were significantly more likely also to agree that maternity care providers may trust ultrasound above clinical examinations during pregnancy (COR 2.24, CI 1.63–3.09; *n* = 736). When adjusting for whether performing ultrasound or not, the odds ratio increased slightly (adjusted odds ratio (AOR) 2.38, CI 1.71–3.29; n = 736). When also adjusting for health profession, the odds ratio remained unchanged (AOR) 2.38, CI 1.72–3.30; n = 736).

**Table 4:** Health professionals’ responses^a^ to statements about ultrasound by background characteristics and other selected variables

**Background variables**	*Maternity care providers may trust ultrasound above clinical examinations in pregnancy*			*Increasing use of obstetric ultrasound may result in less focus on clinical skills*		
	Agree or strongly agree	Disagree or strongly disagree	p-value^b^	Agree or strongly agree	Disagree or strongly disagree	p-value^b^
	**n (%)**	**n (%)**		**n (%)**	**n (%)**	
**Health professional/workplace (*n*)**	***(905)***			***(902)***		
Physicians in hospitals	125 (49.8)	84 (33.5)	***0.014***	93 (37.3)	112 (45.0)	*0.107*
Midwives/nurses in hospitals	196 (59.0)	109 (32.8)		110 (33.1)	183 (55.1)	
Nurses/midwives in health centres	201 (62.4)	103 (32.0)		132 (41.1)	158 (49.2)	
**Age (*n*)**	***(902)***			***(899)***		
≤ 35 years	319 (60.5)	162 (30.7)	*0.190*	191 (36.5)	268 (51.1)	*0.862*
> 35 years	203 (54.1)	132 (35.2)		141 (37.6)	185 (49.3)	
**Public/Private health care**^c^**(*n*)**	***(902)***			***(899)***		
Public	409 (58.3)	225 (32.1)	*0.869*	269 (38.5)	342 (49.0)	***0.027***
Private	43 (60.6)	21 (29.6)		34 (47.9)	29 (40.8)	
Both public and private	69 (53.1)	49 (37.7)		31 (23.8)	80 (61.5)	
**Public/Private health care**^d^**(*n*)**	***(902)***			***(899)***		
Public	409 (58.3)	225 (32.1)	*0.894*	269 (38.5)	342 (49.0)	*0.380*
Private but also public	112 (55.7)	70 (34.8)		65 (32.3)	109 (54.2)	
**Performing ultrasound**^e^**(*n*)**	***(904)***			***(901)***		
Yes	148 (50.5)	102 (34.8)	***0.001***	104 (35.7)	136 (46.7)	***0.035***
No	374 (61.2)	194 (31.8)		230 (37.7)	317 (52.0)	
**Indicated ultrasound exams in uncomplicated pregnancy**^f^	***(897)***			***(894)***		
≤ 3	356 (57.4)	205 (33.1)	*0.645*	224 (36.3)	322 (52.2)	*0.260*
> 3	162 (58.5)	87 (31.4)		109 (39.4)	126 (45.4)	
**“Ultrasound is safe to use”**^g^**(*n*)**	***(792)***			***(789)***		
Agree or strongly agree	421 (59.4)	225 (31.7)	*0.285*	259 (36.7)	361 (51.1)	*0.961*
Disagree or strongly disagree	45 (54.2)	33 (39.8)		31 (37.3)	44 (53.0)	
**“Pregnant women expect ultrasound at consultation”**^h^**(*n*)**	***(809)***			***(806)***		
Agree or strongly agree	348 (62.5)	150 (26.9)	***0.001***	223 (40.3)	271 (48.9)	*0.134*
Disagree or strongly disagree	121 (48.0)	117 (46.4)		80 (31.7)	147 (58.3)	
	*The use of ultrasound has contributed to medicalisation of pregnancy*					
	Agree or strongly agree	Disagree or strongly disagree	p-value^b^			
	**n (%)**	**n (%)**				
**Health professional/workplace (*n*)**	***(904)***					
Physicians at hospitals	191 (76.1)	25 (10.0)	*0.150*			
Midwives/nurses at hospitals	274 (82.5)	26 (7.8)				
Nurses/midwives at health centres	270 (84.1)	29 (9.0)				
**Age (*n*)**	***(901)***					
≤ 35 years	432 (82.1)	37 (7.0)	*0.171*			
> 35 years	301 (80.3)	42 (11.2)				
**Public/Private health care**^c^**(*n*)**	***(901)***					
Public	563 (80.4)	61 (8.7)	*0.811*			
Private	58 (81.7)	8 (11.3)				
Both public and private	111 (85.4)	11 (8.5)				
**Public/Private health care**^d^**(*n*)**	***(901)***					
Public	563 (80.4)	61 (8.7)	*0.457*			
Private but also public	169 (84.1)	19 (8.5)				
**Performing ultrasound**^e^**(*n*)**	***(903)***					
Yes	225 (76.8)	29 (9.9)	***0.002***			
No	509 (83.4)	51 (8.4)				
**Indicated ultrasound exams in uncomplicated pregnancy**^f^	***(896)***					
≤ 3	502 (81.1)	60 (9.7)	*0.200*			
> 3	227 (81.9)	18 (6.5)				
**“Ultrasound is safe to use”**^g^**(*n*)**	***(791)***					
Agree or strongly agree	584 (82.5)	59 (8.3)	*0.174*			
Disagree or strongly disagree	64 (77.1)	12 (14.5)				
**“Pregnant women expect ultrasound at consultation”**^h^**(*n*)**	***(808)***					
Agree or strongly agree	467 (83.8)	46 (8.3)	*0.388*			
Disagree or strongly disagree	198 (78.9)	26 (10.4)				

^a^All five categories of response were included as separate categories in analysis: “Strongly agree”, “Agree”, “Neutral”, “Disagree”, “Strongly disagree”. Response option “neutral” not presented in the table

^b^Pearson’s Chi-Square test for comparison of difference between categories

^c^Included in analysis are only participants who reported working either in public or private health care

^d^Included in analysis are participants who reported working either in public health care solely or working in both public and private health care

^e^Performing ultrasound examinations

^f^Responses to question: “In your view, how many ultrasound examinations are medically indicated in an uncomplicated pregnancy”?

^g^Pre-specified statement: “Ultrasound is safe to use for the pregnant woman and the fetus irrespective of the number of examinations”. Nine hundred and three participants responded to this question. Results for this statement have been previously published [1]

^h^Pre-specified statement: “Do you feel that pregnant women expect to have an ultrasound during consultations, even when there is no medical indication for ultrasound?”

### Increasing use of obstetric ultrasound may result in less focus on clinical skills

Half of all participants (50.2%) disagreed with this statement, while 37.1% agreed (Table 4). The background variables private/public health category and whether performing ultrasound, were associated with the statement (*p* = 0.027 and *p* = 0.035, respectively). When excluding the response option “neutral”, none of the significant associations in Table 4 remained significant.

### The use of ultrasound has contributed to medicalisation of pregnancy

The vast majority (88.5%) of participants agreed with this statement, while a minority (8.1%) disagreed (Table 4). Whether performing ultrasound or not was associated with the statement (*p* = 0.002), but when the response option “neutral” was excluded, the association did not remain statistically significant (Table 4).

**Fig. 1:**
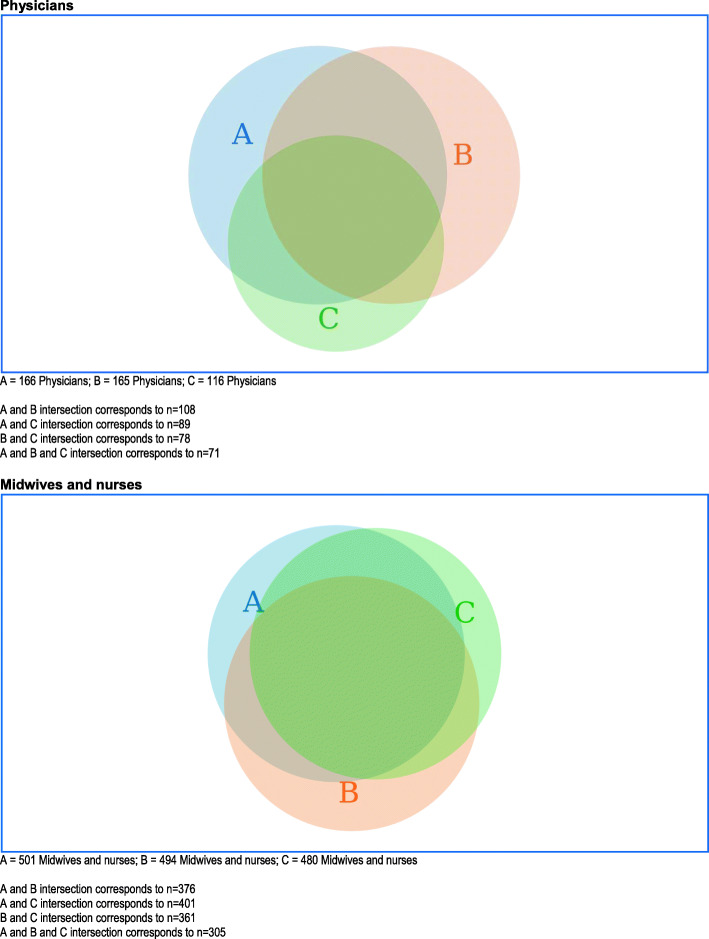
Venn diagrams presenting the numbers of all physicians and midwives/nurses who either agreed/strongly agreed or disagreed/strongly disagreed (specified) with the following three statements: disagreed or strongly disagreed with “Obstetric ultrasound are often performed for non-medical purposes in my country” (**A**; blue area), agreed or strongly agreed with “Pregnant women should be able to have non-medical ultrasound on their own request” (**B**; beige area), and disagreed or strongly disagreed with “Commercialisation of ultrasound is a problem in my country” (**C**; green area)

**Fig. 2:**
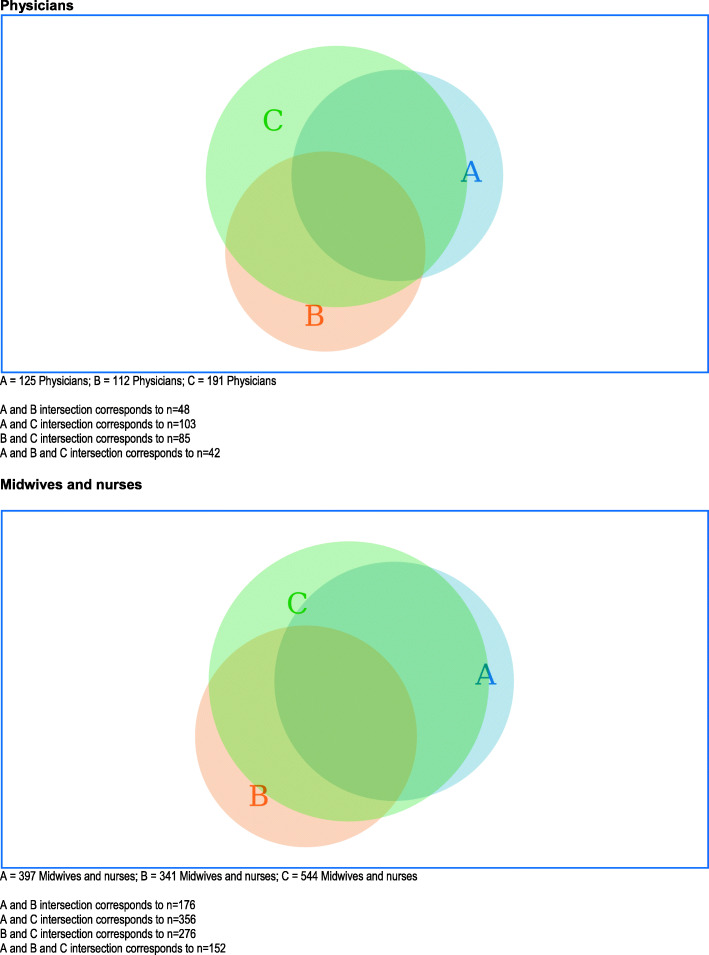
Venn diagrams presenting the numbers of all physicians and midwives/nurses who either agreed/strongly agreed or disagreed/strongly disagreed (specified) with the following three statements: agreed or strongly agreed with “Maternity care providers may trust ultrasound above clinical examinations in pregnancy” (**A**; blue area), disagreed or strongly disagreed with “Increasing use of obstetric ultrasound may result in less focus on clinical skills” (**B**; beige area), and agreed or strongly agreed with “The use of ultrasound has contributed to medicalization of pregnancy” (**C**; green area)

The Venn diagrams (Figs. 1 and 2) illustrate the proportions of agreement/disagreement with different statements presented for the two categories “physicians” and “midwives and nurses”. Figure 1 demonstrates the proportions disagreeing with the statement “*Obstetric ultrasounds are often performed for non-medical purposes in my country*” (statement A), agreeing with the statement “*Pregnant women should be able to have a non-medical ultrasound on their own request*” (statement B), and disagreeing with the statement “*Commercialisation of ultrasound is a problem in my country*” (statement C). For midwives and nurses, the diagram demonstrates a more coherent agreement/disagreement pattern compared with the pattern representing physicians. For example, among midwives and nurses who disagreed with statement A, 75% (376/501) also agreed with statement B. The corresponding figures for agreeing with statement B and disagreeing with statement A was 76.1% (376/494). For physicians, of those who disagreed with statement A, a lesser proportion of 65.0% (106/166) agreed with statement B, and the proportion agreeing with statement B and disagreeing with statement A demonstrated an almost equal proportion of 65.4% (108/165).

Figure 2 shows the proportions agreeing or strongly agreeing with the statement “*Maternity care providers may trust ultrasound above clinical examinations in pregnancy*”, that disagreed with the statement “*Increasing use of obstetric ultrasound may result in less focus on clinical skills*”, and agreed with the statement “*The use of ultrasound has contributed to medicalisation of pregnancy*”. The two diagrams representing physicians and midwives and nurses were similar in their patterns of opinions.

## Discussion

Clinical guidelines for the use of obstetric ultrasound in Rwanda seem to be sparse at any level of the health care system. Fewer than half the physicians answered the question about whether clinical guidelines were adhered to, and of these fewer than half reported that guidelines at their workplace were followed “to a moderate” or “to a great extent.” The Rwandan maternal health care system may benefit from clinical guidelines which state clear medical indications for obstetric ultrasound, as well as allocating scarce resources to areas of need. An alternative explanation may be that guidelines do exist, but knowledge of these clinical guidelines was low among the participants. In either case, there is a clear need for further professional development in this area to enhance evidence-based practice.

In a previous publication from this Rwandan CROCUS study we reported that fewer than half of the participating physicians (44.2%) believed there were sufficient resources to provide medically indicated obstetric ultrasound examinations to pregnant women who needed them [8]. As expected, most participants (74.0%) did not believe that obstetric ultrasound examinations were performed often for non-medical purposes. Most did agree however, that pregnant women expect to have an ultrasound during consultations, even when there is no medical indication for ultrasound. Physicians in hospitals were more likely to agree with this statement compared with midwives/nurses in hospitals, as did participants performing ultrasound compared with those not performing ultrasound. The explanation for this difference between physicians and midwives/nurses is probably related to the situations when pregnant women consult physicians, who are able to perform or order an ultrasound examination if needed. A somewhat surprising result, considering restricted obstetric ultrasound resources in Rwanda, was that almost three quarters thought that pregnant women should be able to have an ultrasound on request and without any medical indication. It is well acknowledged worldwide that pregnant women like having ultrasound examinations during their pregnancy consultations, and that ultrasound examinations may enhance bonding with the fetus [25]. In a previous publication from this study sample, 79% agreed that ultrasound is important for expectant parents to bond with their fetus during pregnancy [8]. There are other important aspects to be considered however, when it comes to obstetric ultrasound examinations without medical indication, such as ethical issues and unnecessary fetal energy exposure. An analysis of non-medical fetal ultrasound concludes that obstetric ultrasound practice is only ethically justifiable if the indication is based on medical evidence [26]. Obstetric ultrasound also entails an energy exposure directed to fetal tissues, and it is well established that the fetus may be negatively impacted by ultrasound energy exposure [27–29]. Therefore, the ALARA principle, i.e. As Low As Reasonable Achievable principle, should always be applied in order to avoid unnecessary fetal energy exposure [27]. The awareness of the potentially negative consequences of fetal ultrasound energy exposure seemed low in this study, since the majority of participants agreed that “ultrasound is safe to use for the pregnant woman and the fetus irrespective of the number of examinations (previously reported) [8]. For most of the participants, commercialisation of ultrasound was not considered to be an issue in Rwanda, either at national or hospital/clinic level. This may be due to the currently restricted access to obstetric ultrasound by private enterprise, and/or, that many participants did not believe there was a medical risk associated with the number of ultrasound examinations. Participants who themselves performed ultrasound were less likely however, to disagree with the statement.

The mean number of ultrasound examinations judged to be medically indicated in an uncomplicated pregnancy was 3.2 in the sample overall, whereas it was significantly higher among physicians in hospitals (3.7) than among midwives/nurses in hospitals (3.3). This difference may be attributed to several factors, among them the lack of, or lack of awareness of, clinical guidelines. There is substantial global variation in national recommendations on the number of ultrasound examinations in an uncomplicated (normal) pregnancy. For example, medical authorities in Norway recommended one routine ultrasound examination at the time we performed the CROCUS study in Norway (2016), where a majority of all participants (59%) were satisfied with the recommended one ultrasound examination, whereas participants using ultrasound themselves were significantly likely to want to offer two or more ultrasounds [30]. In Vietnam, the number of routine ultrasound examinations in an uncomplicated pregnancy, recommended by the Ministry of Health at the time of our CROCUS data collection in the Hanoi area (2017) was three, whereas the Vietnamese participants suggested as many as 5.9 ultrasound examinations were motivated during an uncomplicated pregnancy [31]. Since 2016, the World Health Organization recommends one routine ultrasound examination before 24 weeks of gestation [1]. In a setting such as Rwanda, where access to ultrasound examinations is currently restricted, it is important that ultrasound resources are allocated where they will contribute to the best possible health outcomes.

Most participants agreed that maternity care providers may trust ultrasound above clinical examinations during pregnancy, with only around one third disagreeing. From this single statement we cannot conclude whether they believed this development to be positive or not. Further, participants who agreed that “*Pregnant women expect to have an ultrasound during consultations, even when there is no medical indication for ultrasound*” were more likely to agree that ultrasound may be trusted over clinical examinations. This interrelation may possibly be explained by their somewhat positive attitude towards an overall increase in the use of obstetric ultrasound. Half disagreed however, with the statement “*Increasing use of obstetric ultrasound may result in less focus on clinical skills”*. Figure 2 demonstrates that a minority of physicians agreed that “*Maternity care providers may trust ultrasound above clinical examinations in pregnancy”* and also disagreed with the statement “*Increasing use of obstetric ultrasound may result in less focus on clinical skills”*.

Most participants agreed that “*The use of ultrasound has contributed to medicalisation of pregnancy”*. The concept of medicalisation emerged during the 1970s and 1980s, and in early formulations was considered as “a general trend which involved extension of medicine’s jurisdiction over erstwhile “normal” life events and experiences, as these became categorised as problems appropriate for medical supervision and intervention” [32, 33], among them childbirth [33]. In high-income settings medicalisation is often considered as possibly having a negative impact on maternal pregnancy experiences [33]. An alternative interpretation of the results of our study, is that in the Rwandan context, characterised by scarce obstetric ultrasound and other technical resources for pregnancy surveillance, the term medicalisation might be viewed positively, indicating a technical improvement within the Rwandan health care system.

### Methodological considerations

The aim of this national study was to obtain a representative sample of health professionals working in health facilities in contemporary Rwanda in order to further investigate the findings of previous qualitative research [9, 10]. The study included health professionals currently working at hospitals and health centres across Rwanda. All provincial and referral hospitals were included in the study, as well as a majority of district hospitals. Further, the study included approximately two thirds of all obstetricians/gynecologists, one third of all physicians and one third of all midwives currently working in Rwanda. For participants working in hospitals in Rwanda, we believe that the sample is largely representative. The composition of the total sample cannot be considered however, to be fully representative for the whole country. The study questionnaire was developed following extensive prior qualitative investigation, and its creation and evaluation have been described in detail previously [8].

## Conclusions

Although access to obstetric ultrasound is limited in Rwanda, it is highly valued by health professionals as an important pregnancy surveillance tool. Our findings indicate that clinical guidelines for the use of obstetric ultrasound are limited in Rwanda. Non-medically indicated obstetric ultrasound was not considered a current problem at any level of the health system. Indeed, an unexpected finding was how many health professionals were positive about non-medical obstetric ultrasound examinations on maternal request, something which likely puts a further burden on a maternal health care system with limited resources. It is essential that limited obstetric ultrasound resources are allocated where they are most beneficial, and clearly stated medical indications for obstetric ultrasound examinations would likely facilitate this.

## Data Availability

Data will be available on reasonable request.
